# Supplementary material for: The interplay between vapour, liquid, and solid phases in laser powder bed fusion
Source: Nat Commun. 2022 May 26;13:2959. doi: 10.1038/s41467-022-30667-z (PMC9135709; doi:10.1038/s41467-022-30667-z)
Supplement: Supplementary file 1 — Supplementary Information [file 41467_2022_30667_MOESM1_ESM.pdf]

Supplementary Information for “The interplay between vapour, liquid, and solid phases in laser powder bed fusion”

I. Bitharas et al.

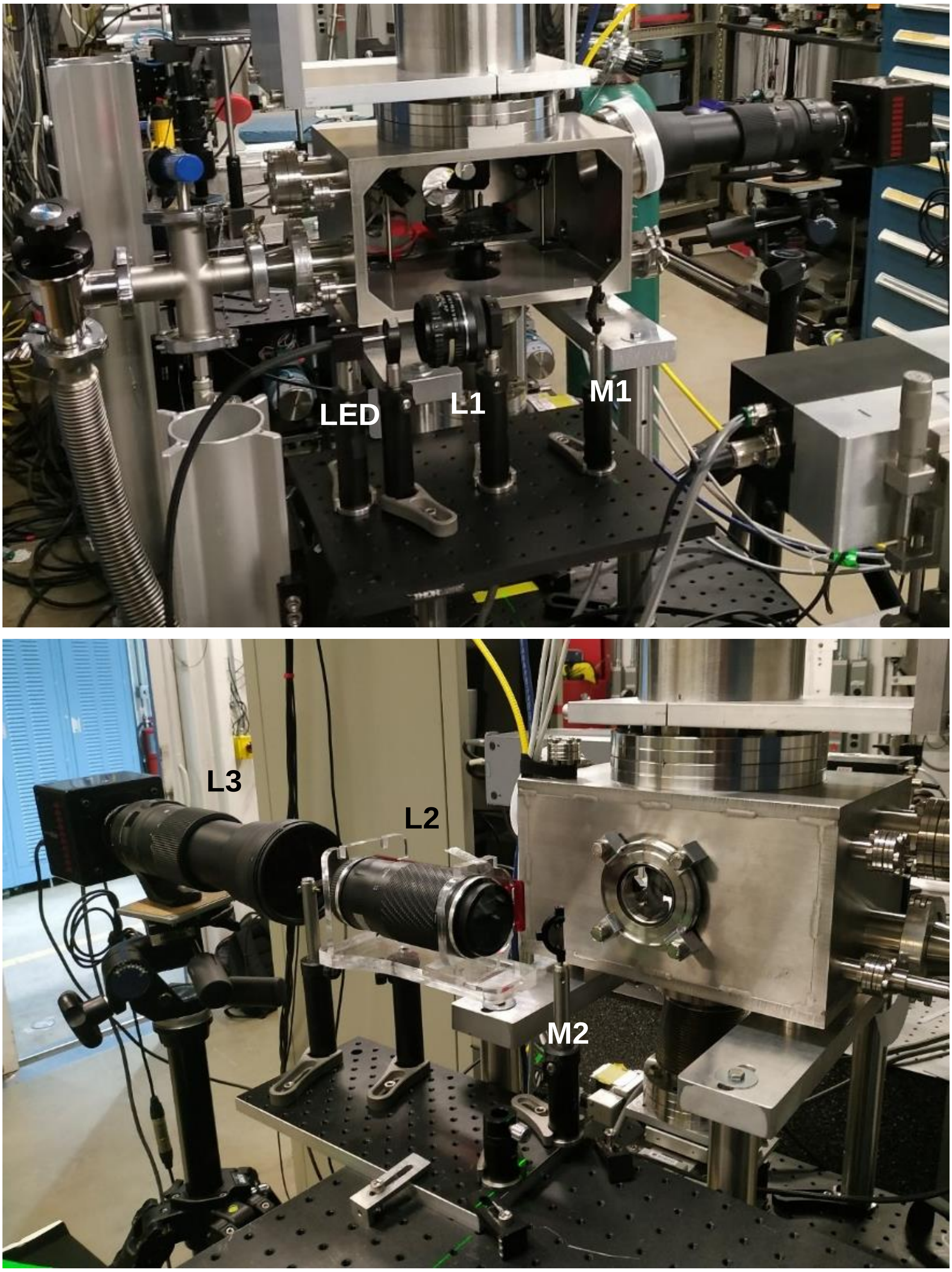

Supplementary Fig. 1. Photographs of the LPBF process simulator with schlieren and x-ray setups at the 32-ID-B beamline of the Advanced Photon Source. : **L1** – SMC Pentax-A 50 mm F1.7, **L2** – Sigma DL 75-300 mm, **L3** – Sigma 150-600 mm f/5-6.3, **M1, M2–** pickoff mirrors.

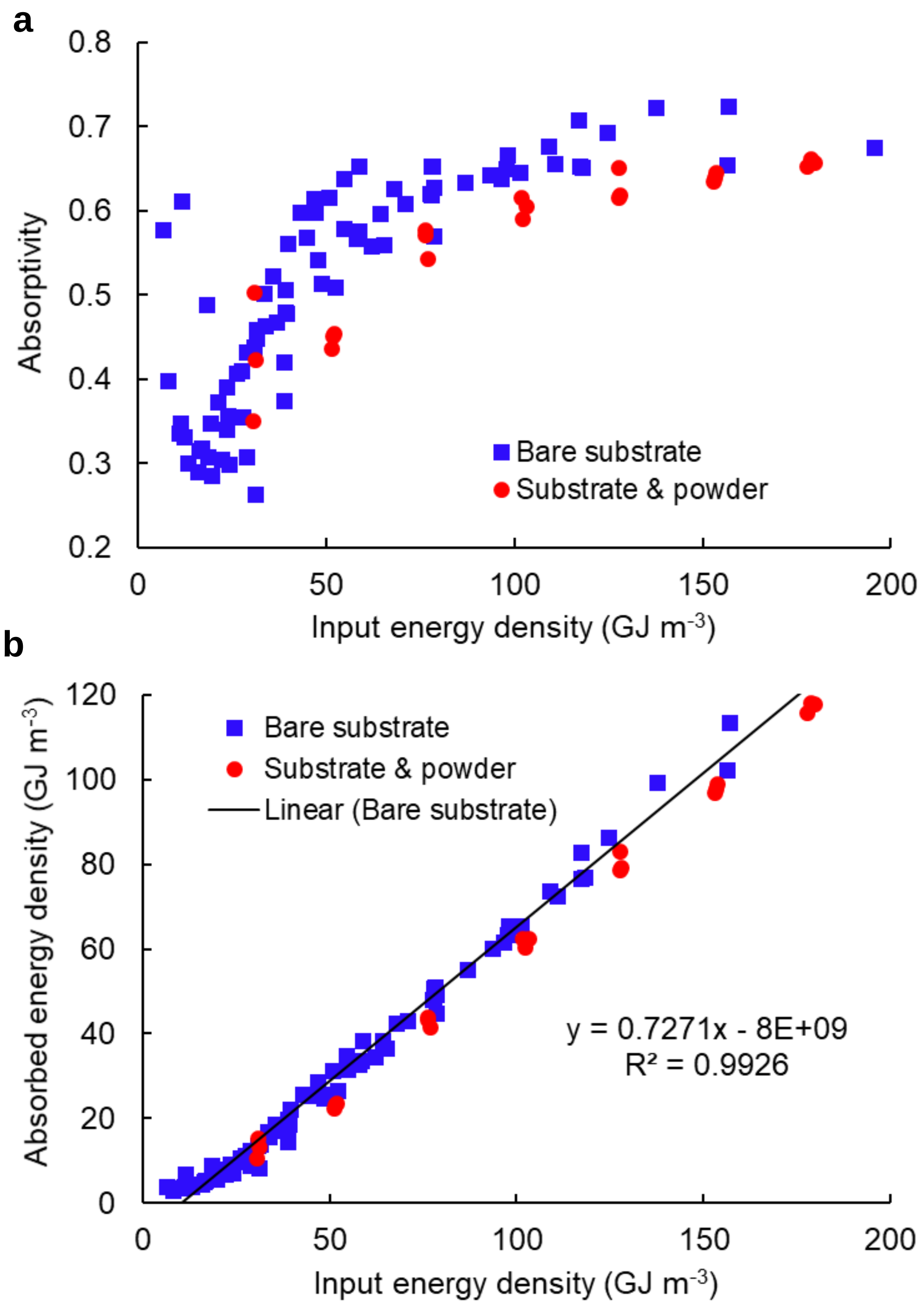

Supplementary Fig. 2. Time-averaged absorptivity measurements for Ti-6Al-4V from Ye et al.[32] (Figure 1, A – C), plotted according to input energy density E. **a** Absorptivity A variation: for 15 < E < 50 GJ m^{-3}, A doubles from 0.3 to 0.6, but it only increases by 16 % for 50 < E < 220 GJ m^{-3}. At low energy input, the powder layer thickness is important but becomes a second-order effect for E > 50 GJ m^{-3}. **b** Absorbed input energy density EA versus E. A linear increase in EA suggests that the depression progressively absorbs more energy as it grows larger.

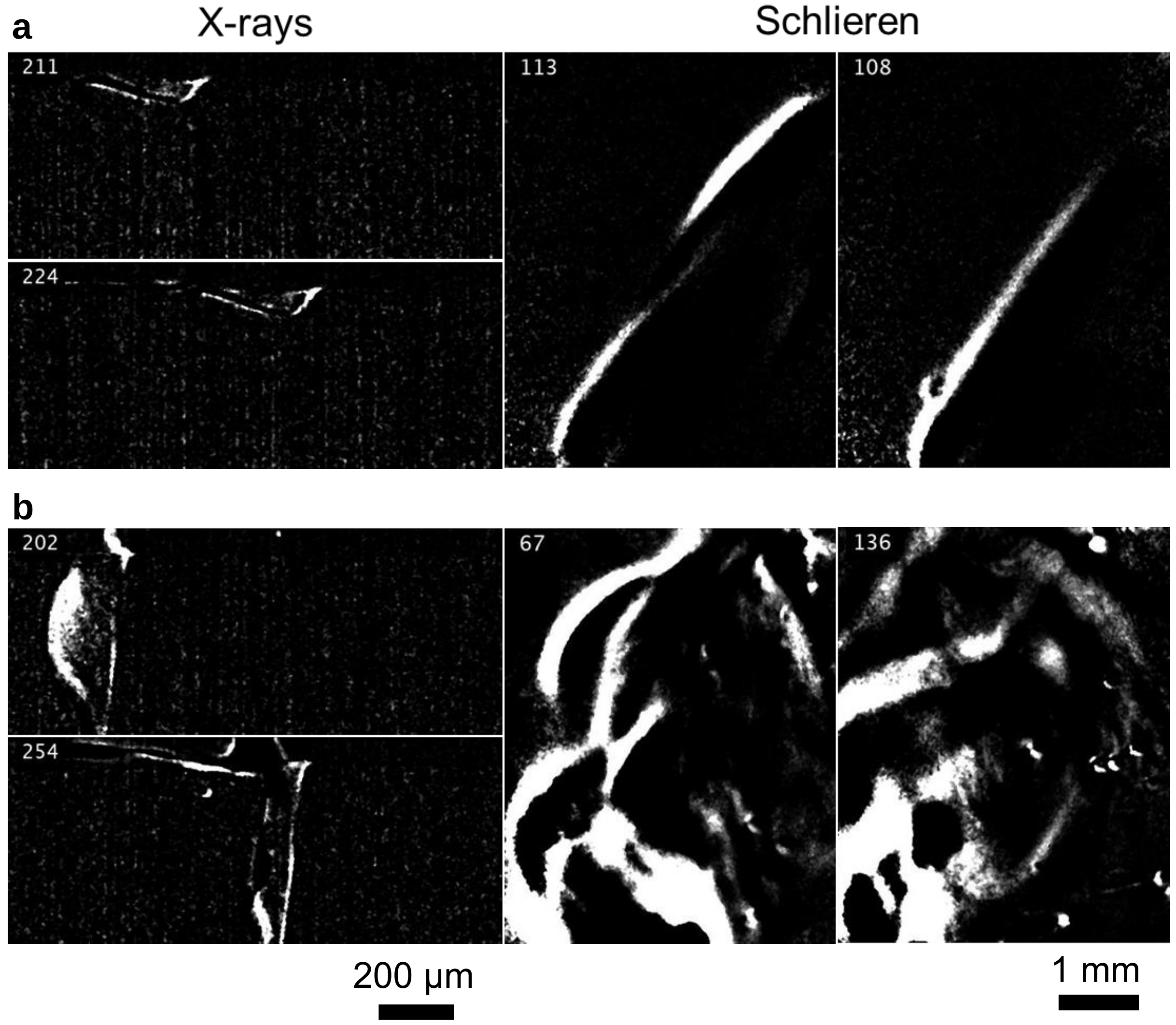

Supplementary Fig. 3 Typical time differenced frames. Each pixel takes the value p_i defined as $p_{i,k} = \left(X_{i,k} - X_{i-1,k}\right)^2$, where X_i is the intensity value of the k-th pixel in the i-th frame of the sequence (i is labelled on the top left of each image). **a** Stage II depression: 210 W, 1 m s^{-1} (Φ = 2.7 MW cm^{-2} E = 27.2 GJ m^{-3}). **b** Stage III depression: 440 W, 0.45 m s^{-1} (Φ = 7.9 MW cm^{-2} E = 177.2 2 GJ m^{-3}). 200 µm scale bar applies to X-ray images, 1 mm scale bar applies to schlieren images.

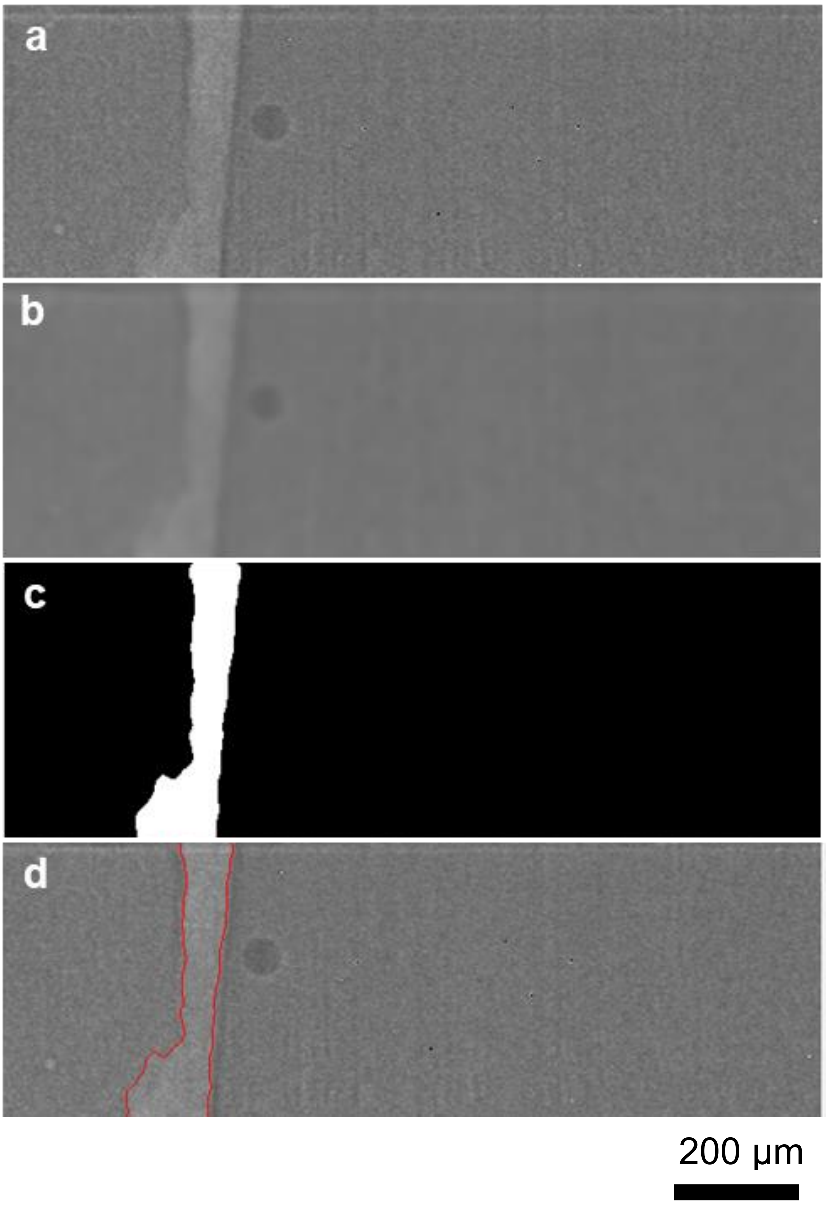

Supplementary Fig. 4. Steps to keyhole boundary detection. a Region of interest on original image. **b** Image after median filtering (7x7 kernel) and Gaussian blurring (σ = 3). **c** Binary image generated (threshold ~0.49). **d** Original (cropped) image with overlaid edge, detected on binary image via the Sobel operator.

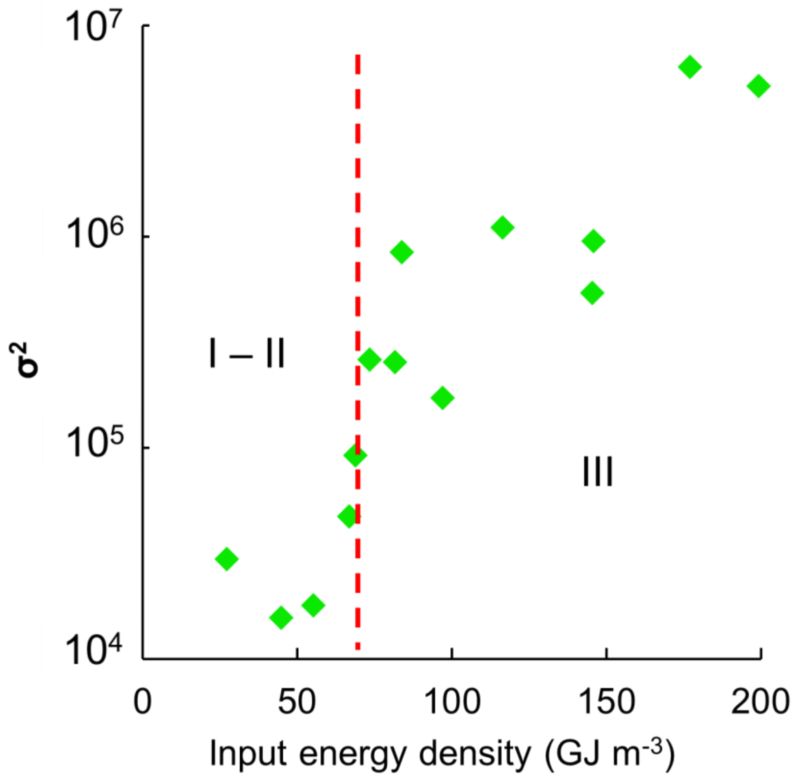

Supplementary Fig. 5. Plot of measured depression variance vs input energy density. The variance of the detected area was calculated as $\sigma^2 = (A - \mu)^2/(N - 1)$, where A is the keyhole area in each frame, and μ is the mean area over N analysed frames.

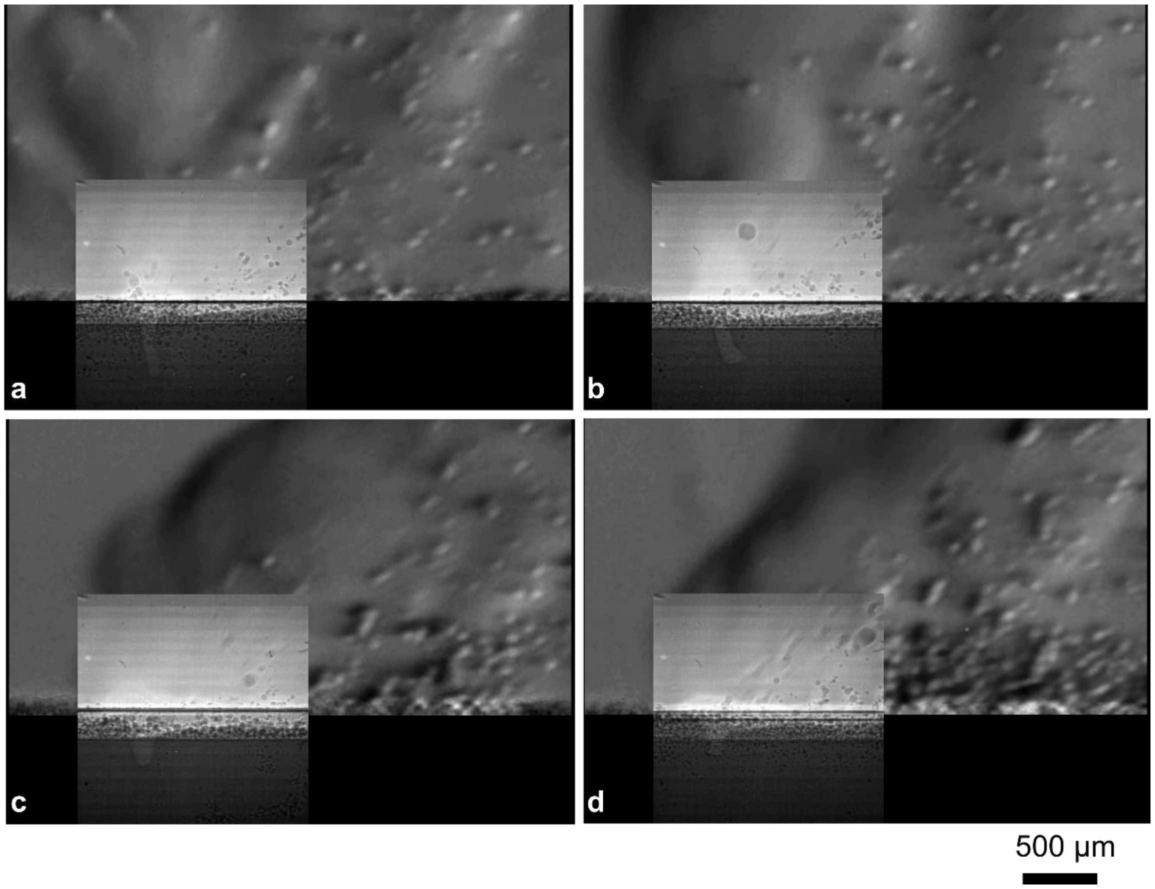

Supplementary Fig. 6. Composite images of line scans with powder at constant laser power P = 322 W, diameter d = 84 µm and varying scan speed u. a Input energy density E = 145 GJ m^{-3}, u = 0.4 m s^{-1}. **b** E = 97 GJ m^{-3}, u =0.6 m s^{-1}. **c** E = 72 GJ m^{-3}, u = 0.8 m s^{-1}. **d** E = 58 GJ m^{-3}, u = 1 m s^{-1}.

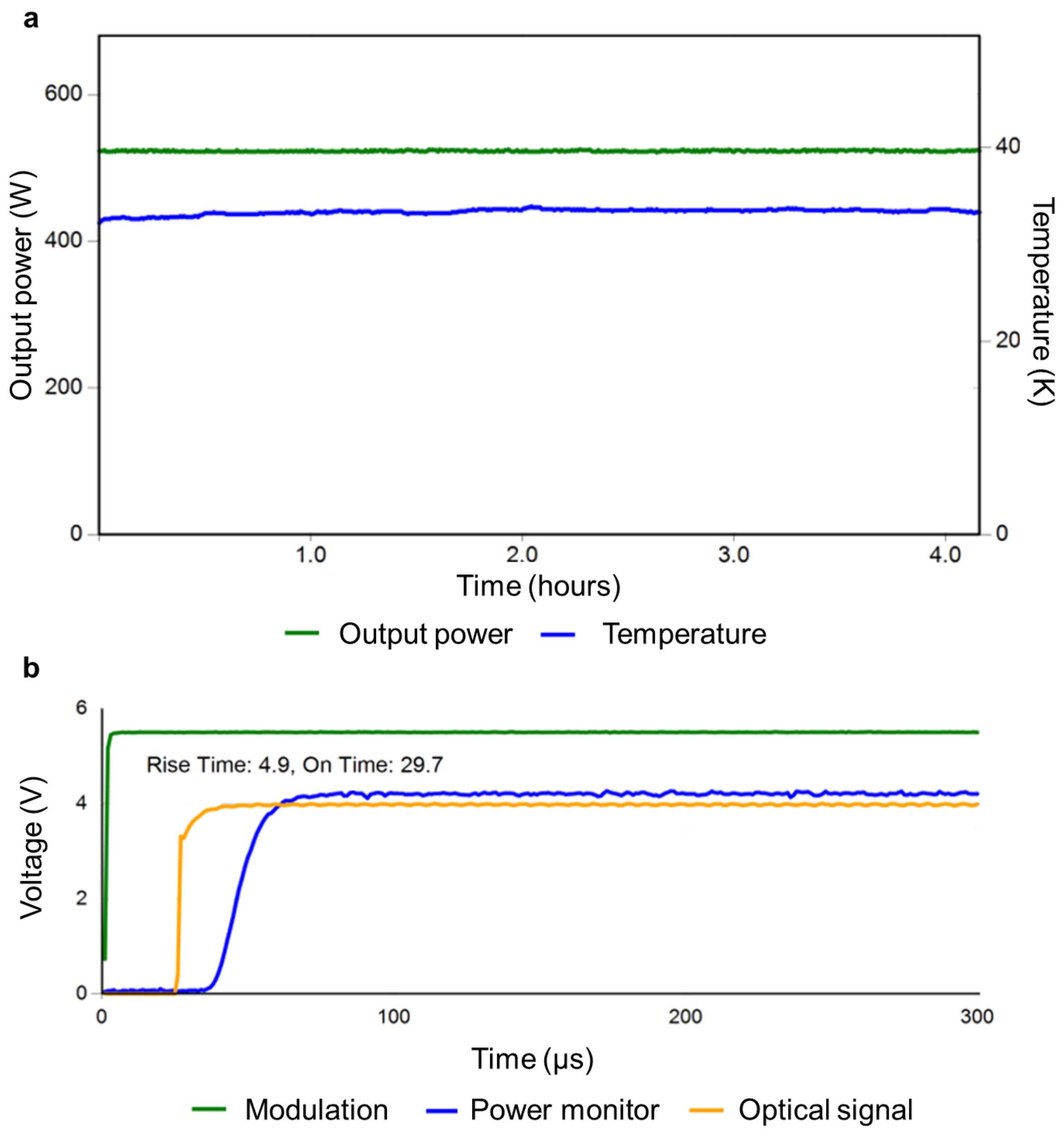

Supplementary Fig. 7. Laser calibration graphs, as measured by the manufacturer (YLR-500-AC-Y11, IPG Photonics). a The output power stability was calculated at 0.4 % after power measurements over 4 hours, with a theoretical maximum of 3% possible for this model. **b** The time taken to reach maximum power was measured to be ~30 μs, which is typical for a fibre laser of this type.
