# Supplementary material for: The interplay between vapour, liquid, and solid phases in laser powder bed fusion
Source: Nat Commun. 2022 May 26;13:2959. doi: 10.1038/s41467-022-30667-z (PMC9135709; doi:10.1038/s41467-022-30667-z)
Supplement: Supplementary file 3 — Description of Additional Supplementary Files [file 41467_2022_30667_MOESM3_ESM.pdf]

**Description of Additional Supplementary Files**

File Name: Supplementary Movie 1
Description: Video for Fig. 2: evolution of vapour depression and plume stability over time under a stationary laser beam.

File Name: Supplementary Movie 2
Description: Video for Fig. 4: depression and plume dynamics with varying input energy density under a scanning laser beam.

File Name: Supplementary Movie 3
Description: Video for Fig. 8 and Fig. 9: comparison of process dynamics with and without powder, at high and low input energy density under a stationary laser beam.

File Name: Supplementary Movie 4
Description: Video for Fig. 10: comparison of process dynamics with and without powder, at varying input energy density under a scanning laser beam.
